# Brazilian Adults' Sedentary Behaviors by Life Domain: Population-Based Study

**DOI:** 10.1371/journal.pone.0091614

**Published:** 2014-03-11

**Authors:** Grégore I. Mielke, Inácio C. M. da Silva, Neville Owen, Pedro C. Hallal

**Affiliations:** 1 Postgraduate Program in Epidemiology, Federal University of Pelotas, Brazil; 2 Baker IDI Heart and Diabetes Institute, Melbourne, Australia; Tokyo Institute of Technology, Japan

## Abstract

**Background:**

There is rapidly-emerging evidence on the harmful health effects of sedentary behaviors. The aim of this paper was to quantify time in sedentary behaviors and document socio-demographic variations in different life domains among adults.

**Methods:**

A population-based survey was carried out in 2012 through face-to-face interviews with Brazilian adults aged 20+ years (N = 2,927). Information about time spent sedentary in a typical weekday was collected for five different domains (workplace, commuting, school/university, watching TV, and computer use at home). Descriptive and bivariate analyses examined variations in overall and domain-specific sedentary time by gender, age, educational attainment and socioeconomic position.

**Results:**

On average, participants reported spending 5.8 (SD 4.5) hours per day sitting. The median value was 4.5 (interquartile range: 2.5–8) hours. Men, younger adults, those with higher schooling and from the wealthiest socioeconomic groups had higher overall sedentary scores. TV time was higher in women, older adults and among those with low schooling and socioeconomic position. Sedentary time in transport was higher in men, younger adults, and participants with high schooling and high socioeconomic position. Computer use at home was more frequent among young adults and those from high socioeconomic groups. Sitting at work was higher in those with higher schooling and from the wealthiest socioeconomic groups. Sedentary behavior at school was related inversely to age and directly to schooling.

**Conclusion:**

Patterns of sedentary behavior are different by life domains. Initiatives to reduce prolonged sitting among Brazilian adults will be required on multiple levels for different life domains.

## Introduction

Changes in transport systems, industrial production modes, innovations in domestic and workplace communication and labor-saving technologies and reorientation of many aspects of the built environments of cities have led to reduction in the energy required to perform the tasks of everyday life.[1] For the past several decades, research and public health initiatives have focused mainly on exercise training and on moderate-to-vigorous intensity physical activity, highlighting its strong association with non communicable disease. [2] However, adults can spend less than 5% of the time awake in a typical day on moderate-to-vigorous physical activity. [3] Most of the time is spent either in light-intensity physical activity or in sedentary behavior. For a comprehensive public health approach, it is also essential to understand the health consequences of what people do in the remaining 95% of their waking hours.

It was only in the last decade that researchers started focusing on activities that require low amounts of energy as light-intensity physical activity and sedentary behavior. Sedentary behavior, i.e. the time spent in activities of 1.5 METs or lower, [4] has attracted widespread scientific attention in recent years. In this perspective, failing to achieve the public health goals on physical activity is not the same as being sedentary, which is usually expressed as sitting time.[5] Recent studies have evaluated the health consequences of sedentary behaviors, showing associations with all-cause mortality [6] and other outcomes [7], [8].

In light of the emerging importance of sedentary behavior for health outcomes, there is the need for descriptive epidemiology findings that can guide public health approaches. For example, Clark et al. examined TV time in a sample of Australian adults and showed that about 46% of men and 40% of women spent two hours per day or more watching TV.[9] Participants of the National Health and Nutrition Examination Survey in the USA spent, on average, 7.7 hours/day in sedentary behavior. [10] Data from 66 countries show that 41.5% of the adults worldwide spend four or more hours per day sitting.[11] A study including information from 20 countries reported a median of 300 minutes per day of sitting time.[12] However, the majority of findings so far reported have been derived from studies in high-income countries. Therefore, there is a need of studies in low- and middle-income countries, particularly because the patterns of sedentary behavior are likely influenced by variations in social, cultural and economic contexts [12].

Most surveillance systems currently operating do not include standardized questions on sedentary behavior. Available international-comparative findings are based on a broad indicator of sedentary time, from a single question on time spent sitting per day.[12] These approaches can underestimate total sedentary behavior. Moreover, an important issue is that – similar to physical activity – sedentary behaviors take place in different life domains, primarily work, leisure-time, and commuting.[5] Identifying the prevalence and variations of prolonged sitting time in these domains is not only important for future public health interventions, but also for occupational health, urban planning and transport-related initiatives [13], [14].

Thus, due the limited information on sedentary behavior in different life domains in low-middle income countries, the aim of the present study was to describe sedentary behaviors in a population-based sample of adults living in the South of Brazil, documenting gender, age, education and socioeconomic variations in sedentary time.

## Methods

The study protocol was approved by the Ethics Committee of the Federal University of Pelotas Medical School and written informed consent was obtained from each participant prior to the interview.

This study is part of a multi-purpose health survey conducted in the city of Pelotas, Southern Brazil in 2012. Pelotas is a medium-sized city in the state of Rio Grande do Sul, located in the southern of Brazil (Latitude: 31°46′19″/ Longitude: 2°20′19″), occupying an area of 1,610 Km^2^. In 2010 the population was 328,275 inhabitants, from which 93.3% live in the urban area. A population-based cross-sectional study was carried out comprising over 3,500 individuals aged 10 years or more. For this specific analysis, we focus on those aged 20 years or more. A multi-stage sampling scheme was adopted. Each Brazilian city is divided into census tracts by the Brazilian Institute of Geography and Statistics. Census tracts are delimited geographical areas comprising approximately 300 households each. All 495 urban census tracts from the city were listed and 130 were randomly selected. Within each sampled tract, we systematically selected, on average, 12 households, totaling 1,732 households across the city. In each sampled households, all individuals from the age range of interest were eligible, except those institutionalized and with severe physical (e.g. patient who underwent major surgery in the past month, patient who had a motorcycle accident and lost the movements in the arms and legs) or mental impairment (e.g. severe cognitive impairment, end-stage Alzheimer's disease). Households were visited by trained interviewers. At least three contact attempts in each sampled household were made. No replacement was used.

A face-to-face interview was conducted using a standardized and pretested questionnaire addressing socio-demographic information as well as health behaviors and diseases. For the purposes of the present analyses, we used information on gender (men/women), age (20–29; 30–39; 40–49; 50–59; 60–69; 70+ years), level of schooling (0–4; 5–8; 9–11; 12+ years of education), and family socioeconomic status categorized into five groups according to a standardized protocol [15] from A (wealthiest) to E (poorest).

Sedentary behavior was assessed by interview questions about the time spent sedentary in a usual weekday in five different domains: workplace (for those employed), commuting, school/university (for those studying), watching TV, and computer use at home. For each domain, an initial question was administered on whether or not the respondent was exposed to sedentary behavior in that domain. For example, the question on time of computer use at home was only asked to those who reported having a computer at home. If a respondent was watching TV at the same time as using the computer, this time was counted in both scores (TV viewing and computer use). The instrument was submitted to a seven-day test-retest reliability study in a sample of 78 individuals; the intraclass correlation coefficients and Lin concordance scores were 0.7 or greater for all items and for the total score.

Six different sedentary behavior variables were used: (a) total sedentary time per day – sum of each individual domain score; (b) TV viewing per day; (c) computer use within the household per day; (d) sitting time per day at work; (e) sitting time at school/university per day; (f) time spent sitting inside cars, motorcycles or buses per day. For the total score, those reporting no exposure to a given domain of sedentary time were assigned the value 0 for that specific domain. In domain-specific analyses, however, those not exposed to that source of sedentary time were treated as missing. For example, a respondent with no computer at home was assigned a score 0 for computer time inside the household in the total score, but was excluded from the analyses from that specific domain. This strategy was used to guarantee clear denominators for each analysis.[16]

Descriptive analyses were carried out to check the normality of the data (combined score and specific domains), and to describe total and domain-specific sedentary behavior. Bivariate analyses were then carried out according to gender, age, schooling level and socioeconomic status. Because most scores were asymmetrical, both the mean and the median were used to represent central tendency. We also divided the total score at the median point (270 minutes per day) for categorical analysis. In continuous analyses, K tests for the comparison of medians were used, whereas in categorical analyses, chi-squared tests were employed. Despite data asymmetry, some of our results are presented as means in order to better compare our findings with those from other studies. All analyses were conducted in Stata 11 and took the clustering of the sample into account through the set of commands ‘svy’.

## Results

Within the 1,732 households sampled, we located 3,381 adults aged 20 years or more, of whom 2,927 (86.6%) were interviewed. Most non-respondents were men (56.4%) and no difference in age was detected. In total, 55 were excluded from the analyses due to missing information in one or more of the sedentary behavior variables. The proportion of individuals in the sample practicing 150 or more minutes per week of physical activity was 54.4% (95%CI 51.8; 56.9).

**Figure 1:**
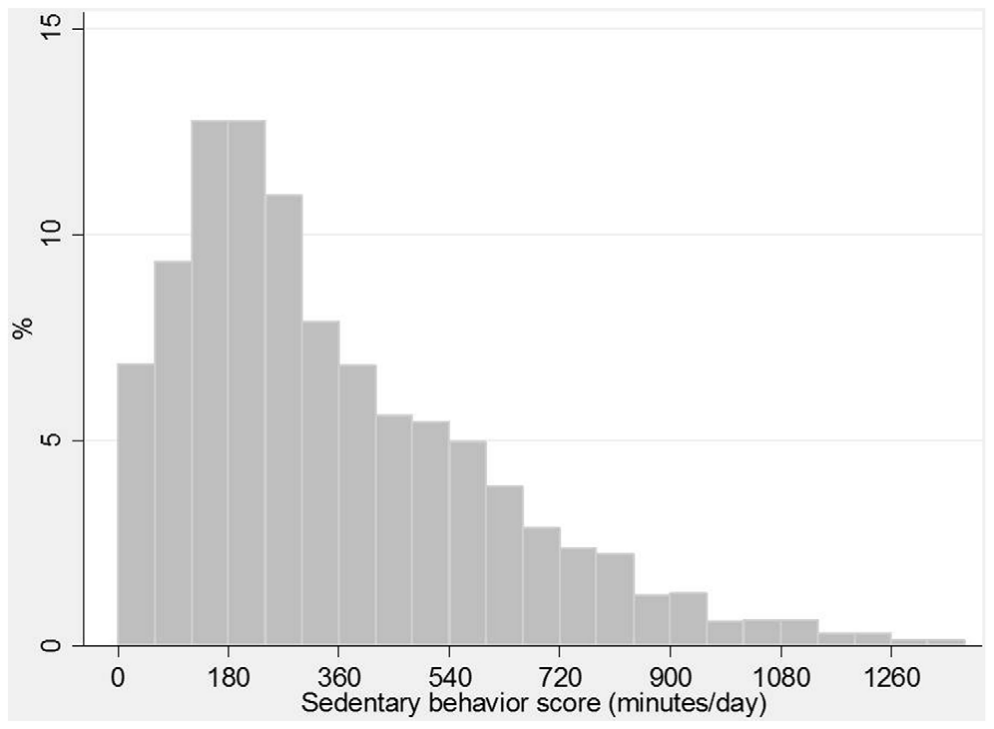
Distribution of the overall sedentary behavior score (minutes per day).

On average, participants spent 345 minutes per day (SD 267) sitting. The median value was 270 with an interquartile range of 150 – 480.The distribution was asymmetrical to the right (Figure 1) with a skewness coefficient of 1.4 and kurtosis of 5.6; 73 participants scored zero in the sedentary behavior score, and the 90^th^ percentile was 720 minutes per day.

Table 1 shows the proportion of the sample in each independent variable subgroup, the mean and median of the sedentary time score in each subgroup and the proportion of the sample with a sedentary score of 270 minutes or more per day. Men, younger adults, those with higher schooling and those from the wealthiest socioeconomic groups had higher sedentary time scores in this unadjusted analysis. The median score was 60 minutes per day higher among men than women; 295 minutes per day higher among those aged 20–29 years as compared to those aged 70 years or more; 330 minutes per day higher among those with 12 years or more of schooling as compared to those with 0–4 years of education; and, 290 minutes higher among those in the wealthiest socioeconomic group as compared to those in the poorest group. All analyses were repeated using Linear and Poisson regression models, but because findings were similar to the unadjusted ones, only those are presented.

**Table 1:** Description of the sample in terms of sociodemographic variables and the associated overall sedentary behavior score (minutes).

Variables	N	%	Mean (CI_95%_)	Median (25–75)	p b	>270 minutes (CI_95%_)	p c
Gender					<0.0001		<0.0001
Men	1,184	41.2	376 (355–397)	300 (180–540)		54.4 (50.3–58.4)	
Women	1,690	58.8	323 (305–341)	240 (135–480)		45.2 (41.8–48.6)	
Age (years)					<0.0001		<0.0001
20–29	607	21.1	476 (446–505)	475 (240–660)		70.3 (65.4–75.3)	
30–39	535	18.6	392 (362–423)	330 (180–540)		56.7 (51.3–62.2)	
40–49	586	20.4	320 (296–344)	240 (135–450)		45.9 (40.9–50.9)	
50–59	507	17.6	311 (284–338)	240 (135–440)		44.2 (38.3–50.1)	
60–69	381	13.3	250 (227–274)	210 (120–320)		33.9 (28.1–39.6)	
70+	258	9.0	200 (179–222)	180 (120–260)		21.4 (16.0–26.8)	
Schooling (years of education)					<0.0001		<0.0001
0–4	503	17.5	190 (172–207)	150 (75–240)		19.5 (15.7–23.3)	
5–8	801	27.9	264 (247–281)	210 (120–340)		35.1 (31.7–39.2)	
9–11	809	28.2	358 (338–377)	300 (180–510)		52.0 (48.2–56.0)	
12+	758	26.4	520 (499-541)	480 (320–685)		80.1 (77.0–83.2)	
Socioeconomic position a					<0.0001		<0.0001
A (wealthiest)	175	6.1	489 (451–526)	465 (300–620)		80.6 (74.3–86.9)	
B	1,155	40.5	397 (377–416)	340 (180–550)		59.3 (55.5–63.0)	
C	1,238	43.4	307 (288–327)	240 (120–420)		40.8 (37.0–44.6)	
D/E (poorest)	285	10.0	205 (178–233)	175 (70–270)		23.2 (17.0–29.4)	
Total	2,927	100.0	345 (328–362)	270 (150–480)		49.0 (45.7–52.2)	

a maximum number of missing values (n = 21);

b K test for the comparison of medians;

c chi-square for heterogeneity.

**Figure 2:**
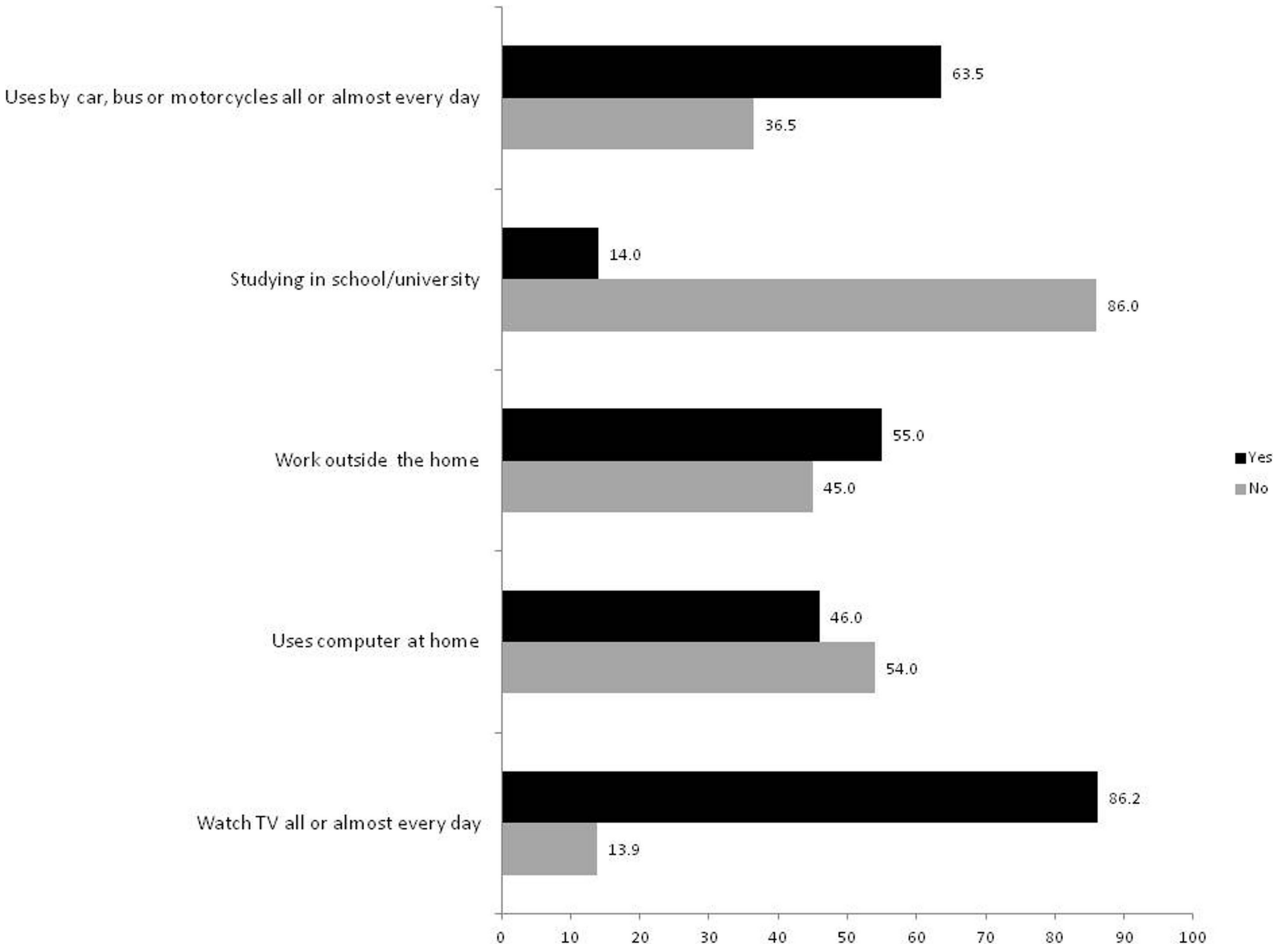
Proportion of respondents exposed to sedentary behavior in each domain.

**Figure 3:**
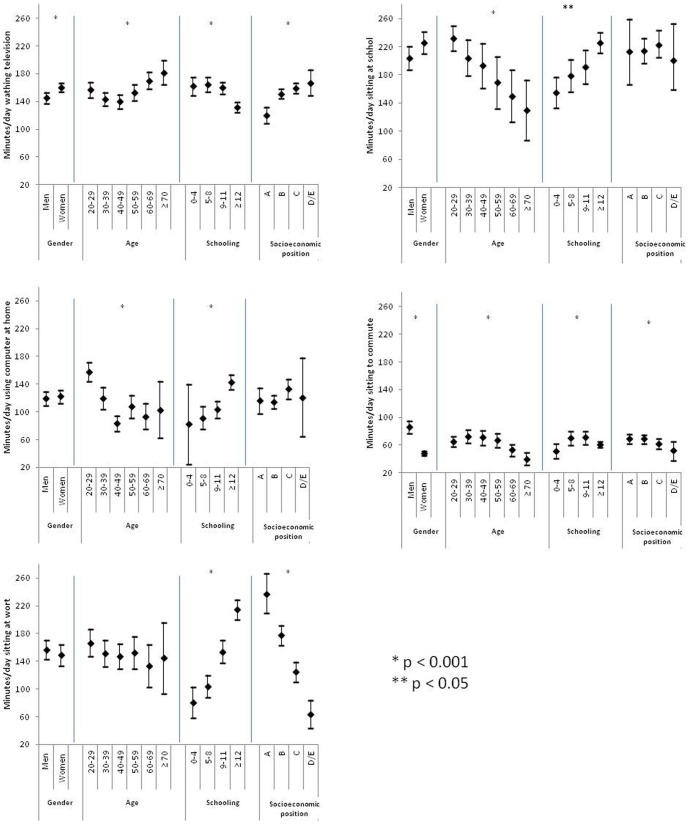
Variations in minutes of sedentary time by gender, age, educational attainment and socio-economic position for different life domains (means and 95% confidence intervals).

Figure 2 presents the proportion of subjects exposed to sedentary time in each of the five domains investigated: 63.5% of the sample reported using cars, buses or motorcycles on an everyday basis; and 86.2% reported watching TV daily. Figure 3 presents the mean (95% confidence interval) of each sedentary time domain in subgroups of the independent variables. For TV viewing time, women had higher levels of sedentary time than did men; older age was associated with higher TV viewing times; and, higher educational attainment and higher socioeconomic position were associated with lower levels of TV viewing time. We tested an interaction term between gender and age based on previous studies, but found no indication of interaction (P = 0.61). Gender was not associated with sedentary time in any of the other domains, except for commuting, in which men had higher levels of sedentary time than did women. Higher educational attainment was associated with higher levels of sedentary time in all other domains. Higher socioeconomic position was related to higher levels of sedentary time the work and commuting domains.

**Figure 4:**
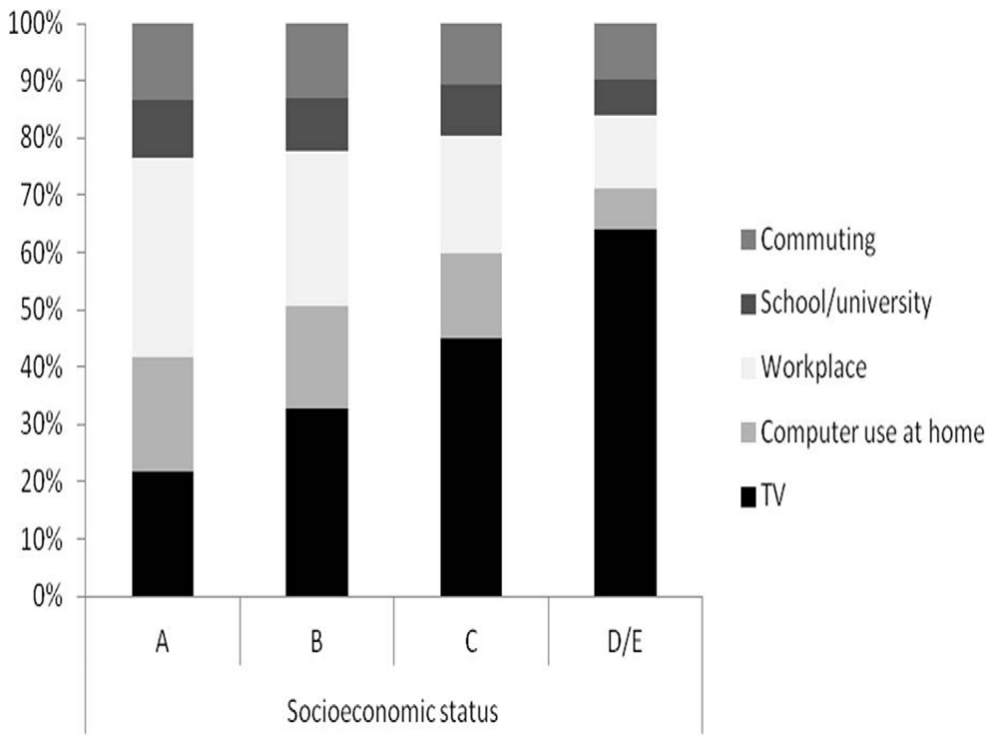
Proportion of total sedentary time by life domains representation (%) according to socioeconomic status.

Figure 4 displays the proportion of total sedentary time by life domains across socioeconomic position categories. The relative importance of watching TV was linearly related to socioeconomic position. In terms of workplace sedentary time, the findings were in the exact opposite direction; high socioeconomic position was related to more sedentary time in the workplace. The same was observed for computer use. No clear patterns were observed for commuting and school/university sedentary time.

## Discussion

Physical inactivity has been characterized as having been the ‘Cinderella’ of risk factors for NCDs,[17] receiving much less scientific and policy attention than required based on its high prevalence [11] and massive negative health consequences.[2] With physical inactivity being recognized as one of the key priorities for public health worldwide, [18] there is the need for a broader understanding of human movement. It is important to now recognize that adults spend on average less than 5% of their waking hours in moderate to vigorous-intensity physical activity.[3] In recent years, a broad body of evidence has begun to demonstrate that how adults spend the remaining 95% of the day also matters a lot for health. [3], [7], [19] For example, those who are obese can spend, on average, two extra hours per day sitting as compared to their non-obese peers.[20] Recent findings suggest that replacing sedentary time with light-intensity activities is likely to be beneficial to health.[7]

Descriptive epidemiology findings on sedentary time have not been reported extensively, particularly findings from low and middle-income countries. Our study is one of the first to present a description of sedentary time in a middle-income setting. These findings also add to the current knowledge by presenting sedentary time data separate for different life domains, namely occupation, transport, study and leisure time. We were unable to validate our measurement tool against accelerometers; our group is currently running such a study. However, our questionnaire presented good reliability.

Some limitations of the present study should be discussed. Despite the wide variety of domains of sedentary behavior reported in the literature, we were only able to study the five that were most often evaluated among adults. We therefore assume our measurement comprises most of the day, but not all of it. In addition, the domains evaluated have high potential in terms of interventions. Some participants reported no sitting (2.5%), while others reported 21–22 hours of sitting per day. Such values are unlikely, but it is important to keep in mind that these values are completely diluted by the large sample size in our descriptive analyses. In fact, population estimates are more important than individual estimates in this case. Lastly, in our sample older participants had lower sedentary time than their younger peers did, which is biologically unlikely. The likely reason is that our questionnaire investigated domains that are more relevant to younger and middle-aged adults than to older adults. Including domains that are more relevant to older adults is a methodological challenge for future studies.

In our sample, patterns of sedentary time varied by life domains. Men had higher levels of sedentary time in the commuting domain, but women had higher TV viewing times. Age was inversely related to sedentary time in most domains, but older adults did have higher TV viewing times. High-income participants were less likely to have high levels of TV viewing time, but it has higher sedentary time scores in the occupational domain. An interesting finding was that the pattern of sedentary time in all other domains tended to differ from the pattern of TV viewing. In addition, TV viewing was inversely related to the total sedentary time score, whereas all other domains were directly associated with the total score. These findings should be highlighted because TV time frequently is often used as a proxy of total sedentary time. These findings should therefore be considered in the future development of research instruments for measuring sedentary time in population-based surveys.

Based on these findings, studies relying solely on TV viewing are likely reporting correlates of sedentary time which are different from what would be observed if a more comprehensive measure of sedentary time was employed. In our sample, taking the total score as the outcome variable, the single component of it that explains the smallest proportion of the variance (10.9%) is TV viewing. The social patterning of screen time was recently evaluated by Stamatakis and colleagues;[21] they found a strong inverse association between socioeconomic deprivation and screen time. TV viewing may be the main leisure activity among the poor due to the lack of other options, whereas among the better-off, the existence of other alternatives may act to reduce TV viewing time and allow a more heterogeneous range of leisure time activities.

Comparing our findings with previous studies is challenging, because the measurement tools tend to vary and the correlates of sedentary time may also vary according to social, cultural and environmental characteristics of samples from different countries. Using data from 20 countries, Bauman and colleagues [12] found no consistent association between gender and sitting time, but reported associations with age and educational attainment that are consistent with those found in our sample. Using data from 66 countries, Hallal and coworkers [11] showed similar sitting time scores between men and women, but higher scores among older adults as compared to those aged 59 years or less.

The social patterning of sedentary time is complex. Those with lower levels of educational attainment and lower family incomes reported lower sitting times. However, one should not interpret this finding as evidence of positive behavior in these groups. In fact, this finding appears to be determined by macro determinants rather than a positive health choice. For example, spending too much time sitting at work may be an indirect indicator of economic success. The same logic may also apply to commuting time in Brazil, where those with higher levels of material well-being may be more likely to have a car and therefore not walk or use other forms of active transportation.

Utilization of information on the descriptive epidemiology of sedentary time has potentially important implications for public health action. Interventions targeting different subgroups of the population can take into account the different life domains in which sedentary time accumulates among these people. For example, among those with higher family incomes, the most likely relevant alternatives are likely to include the promotion of active breaks at work and active transportation. For those with lower incomes, however, campaigns aimed at increasing access to public facilities allowing active time and strategies promoting the involvement in leisure-time physical activity are more likely to succeed. An ecological approach to public health interventions targeting sedentary time [13] would include more than just communicating people about the harmful effects of sitting too much. In fact, it is a key public health priority to help build dynamic societies in which active instead of sedentary activities are encouraged, affordable, safe and valued. [11]
